# Embedding audiological screening within memory clinic care pathway for individuals at risk of cognitive decline—patient perspectives

**DOI:** 10.1186/s12877-021-02701-0

**Published:** 2021-12-14

**Authors:** Anna McDonough, Joshi Dookhy, Cathy McHale, Jennifer Sharkey, Siobhan Fox, Sean P. Kennelly

**Affiliations:** 1 Department of Age-Related Health Care, Tallaght University Hospital, Dublin, Ireland; 2 Department of Audiology, Tallaght University Hospital, Dublin, Ireland; 3 Department of Medical Gerontology, Trinity College Dublin, Dublin, Ireland; 4 Centre for Gerontology and Rehabilitation, University College Cork, Dublin, Ireland

**Keywords:** Cognitive impairment, Hearing, Dementia, Audiology, Brain health

## Abstract

**Background:**

With the evolving knowledge on hearing as a potentially modifiable mid-life risk factor for dementia, identification of people at risk becomes increasingly important. People with mild cognitive impairment (MCI) presenting to specialist memory services represent a key “at-risk” target population for audiological evaluation, but few services have established this pathway. This study sought to examine the patient experience and understanding of this process.

**Methods:**

All patients with MCI attending a tertiary referral memory service referred for audiology review were contacted. A patient survey was delivered over the phone. Outpatient letters and the memory clinic database were reviewed.

**Results:**

Twenty patients with MCI were included in the survey. Eight (8/20, 40%) had self-reported hearing loss. Upon formal audiological assessment seventeen (17/20, 85%) had objective evidence of hearing loss; nine (9/17, 52.9%) with mild-moderate and eight (8/17, 47%) with moderate-severe hearing loss. Only six patients (6/20, 30%) recalled having the rationale behind having a hearing test as part of their memory work-up explained to them. However, the majority (15/20, 75%) felt a hearing test was an important part of their memory assessment. Just seven patients overall (7/20, 35%) identified a link between hearing-loss and cognition. All patients who provided feedback on the service itself made positive comments, although (4/20, 20%) felt they did not get adequate information about the results.

**Conclusions:**

A significant proportion of people with MCI had de-novo evidence of hearing impairment upon assessment. Patients are satisfied with incorporating audiological evaluation into a memory clinic assessment, however clear communication around indication, recommendations, and follow-up ensuring compliance is required.

**Supplementary Information:**

The online version contains supplementary material available at 10.1186/s12877-021-02701-0.

## Background

Much recent research has been focused on interventions to identify and modify the risk factors for dementia, particularly since the publication of the Lancet meta-analysis in 2017 [1] and update in 2020 [2]. The number of people with dementia globally is rising [2], and with increasing knowledge of the risk factors involved, the focus is on disease modification for patients with dementia in the early stage or before symptoms present. Targeting patients early, such as those with mild cognitive impairment (MCI), and optimizing their risk factor profile, could delay or even prevent the development of dementia, with significant personal and societal advantages resulting. Hearing has emerged as the strongest modifiable mid-life risk factor for developing dementia [2]. Furthermore, hearing loss is predicted to be in the top ten causes of disability in higher income countries by 2030 [3], making it an attractive target for screening and intervention.

Both self-reported and informant reports of hearing impairment are associated with cognitive decline [4, 5]. In populations without cognitive impairment, self-reported hearing deficits have a reasonable correlation with objective measurements [6]. However, people with dementia often underreport hearing difficulties, with hearing impairment going undetected in up to 80% [7]. People with dementia often lack insight into their hearing deficits and may have difficulties with audiology assessments and interventions [8]. This highlights the importance of screening for hearing deficits in high-risk populations to identify patients most likely to benefit from intervention. In memory clinics where there is a blanket referral pathway for audiology assessment based on cognitive diagnosis, a significant amount of hearing impairment is identified [9], allowing intervention for an otherwise unidentified, key risk factor for cognitive impairment. With sensory interventions potentially improving not only cognition, but also quality of life and behavioral disturbance [10], auditory screening has the potential to meet a clearly unmet need. However, hearing cannot be examined in isolation. Major public health issues such as hypertension, alcohol excess and obesity [2] are also important mid-life risk factors for dementia and should be addressed to optimize a person’s risk factor profile and brain health.

Hearing impairment can also be associated with under-performance on cognitive testing, one of the hypotheses for its association with cognitive impairment [11]. Many of the commonly used assessments rely on auditory cues and questions, although the minority of physicians actually ask about hearing difficulties [3], therefore potentially undermining the person’s cognitive score if they are unable to fully participate in the evaluation due to hearing loss. Modification of standard cognitive assessments to exclude those reliant on auditory input may somewhat mitigate this deficit [12]. Hearing aid use may also improve cognitive performance by decreasing cognitive load [5] and improving communication [13].

A number of interventions for hearing loss exist, from auditory rehabilitation and listening devices to hearing aids and cochlear implants [14]. It is less clear, however, as to whether such interventions make any difference for patients at risk or diagnosed with a cognitive disorder. Compliance with hearing aid recommendations is another issue, with up to 40% of patients not wearing them [15], making it difficult to determine the optimal dose if hearing deficits are identified [16]. Recently, a number of large, prospective, community-based population studies have suggested that hearing aid use mitigates the higher risk of dementia associated with hearing loss [13, 17, 18], although social isolation potentially mediates some of the risk in hearing aid non-wearers [13].

With relatively low cost and safe options available, screening for hearing impairment is an attractive option for patients with MCI. To our knowledge, just one study to date examines patients’ perception of this type of referral pathway. Wolski et al. [19] describe a focus group and interview based descriptive study of a group of people with dementia and either vision or hearing impairment. This study identified poor understanding following the assessment as to the nature of their deficits and what specific recommendations were made. Patients in this study mostly had a diagnosis of dementia. Patients with MCI, although important as a potential target group for early intervention, were in the minority.

### Study aims

This study sought to explore the patient experience, tolerability and understanding of having an audiology assessment as part of the process of their memory clinic assessment in a group of patients with MCI, and the challenges associated with this pathway. As more research and knowledge around hearing impairment and its links to dementia become available, it will become increasingly relevant how patients feel about audiology testing and intervention.

## Methods

### Setting

This study was carried out in a tertiary referral memory service. This clinic offers a multi-disciplinary approach to cognitive assessment, diagnostics and post-diagnostic support. All patients diagnosed with MCI are offered routine audiology referral since 2018, with the referral made on the basis of a cognitive diagnosis, rather than subjective hearing loss. These patients then attend a separate specialist assessment in the audiology department, lasting approximately 90 min. This involves a full audiological history, otoscope, tympanometry, pure tone audiometry, speech audiometry, with hearing test and Ear Nose and Throat (ENT) referrals made as required.

### Participants

This study included all patients with MCI who agreed to referral for audiology assessment from the memory service. MCI diagnosis was made by expert consensus discussion, following review of extensive cognitive testing, clinical history and examination, collateral history and neuroimaging, and using the National Institute on Aging diagnostic criteria [20].

### Research design and data collection

A patient survey was constructed, based on previous similar studies and aiming to provide a mix of open and closed ended questions to yield both qualitative and quantitative data. The survey used was developed specifically for the purposes of this study (Additional file 1).

Data collection occurred in March and April 2020. All patients with MCI were contacted by phone. Once a consent form was completed, the survey was delivered over the phone by the primary researcher. If the researcher delivering the survey perceived issues with communication by phone, the patient was given the option to withdraw from the study or meet the researcher face-to-face in the hospital.

Further quantitative data was obtained from the memory clinic database and audiology results.

### Data analysis

Due to the mixture of different types of quantitative data, a combination of descriptive statistics and basic inferential analysis was necessary. Statistical analysis looking for correlations between different subgroups was performed. The small amount of qualitative data produced was analysed using thematic analysis [21].

The study protocol was reviewed and approved by the Tallaght University Hospital / St. James’s Hospital Joint Research Ethics Committee.

## Results

**Fig. 1:**
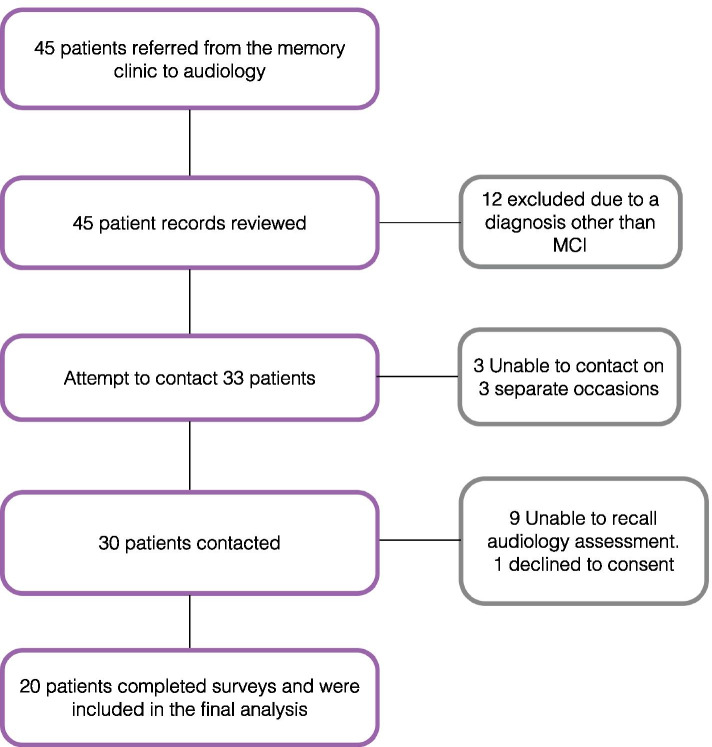
Patient selection process

Between October 2018 and January 2020, forty-five patients agreed to referral from the memory clinic to audiology assessment (Fig. 1), thirty had a diagnosis of MCI.

Those with a diagnosis other than MCI or those who could not recall their audiology assessment were excluded, leaving twenty patients who completed the survey included in the final analysis. They had a mean age of 73.0 (range 57–88) years and just under half (45%) were female. Further demographic information, past medical history, risk factors and cognitive assessment scores are summarised in Table 1.

**Table 1:** Demographic information, risk factors and cognitive assessment scores for included patients

	Mean (Range) or %
Age (Years)	73 (57–88)
Marital status	
Married	85%
Separated	10%
Widowed	5%
Duration of cognitive symptoms (Months)	26.5 (7–54)
Family history of a cognitive disorder	30%
Currently employed	15%
Currently driving	65%
History of alcohol misuse	20%
Smoking status	
Current smoker	15%
Ex-smoker	35%
Never smoked	60%
History of stroke	20%
Hypertension	55%
Type 2 diabetes	15%
Atrial fibrillation	15%
Parkinson’s disease	5%
Ischaemic heart disease	15%
Number of regular medications	5 (0–10)
Number of medications with anti-cholinergic effects	0.7 (0–4)
MMSE	26.4 (21–30)
Clinical Dementia Rating Scale	0.8 (0–2)
Clinical Dementia Rating Scale (Sum of Boxes)	0.7 (0.5–7.5)
AD8 Dementia Screening Interview	3.6 (0–8)

Medical co-morbidities were common, particularly cardiovascular risk factors. Of the twenty patients included, eighteen (18/20, 90%) had at least one of: hypertension, smoking history, type 2 diabetes or history of cardiovascular or cerebrovascular disease. Eleven patients (11/20, 55%) had at least two of these risk factors. Polypharmacy was also prevalent, with a mean and median of 5 regular medications per patient. The mean Mini Mental State Examination (MMSE) score was 26.4 (SD = 2.3).

**Fig. 2:**
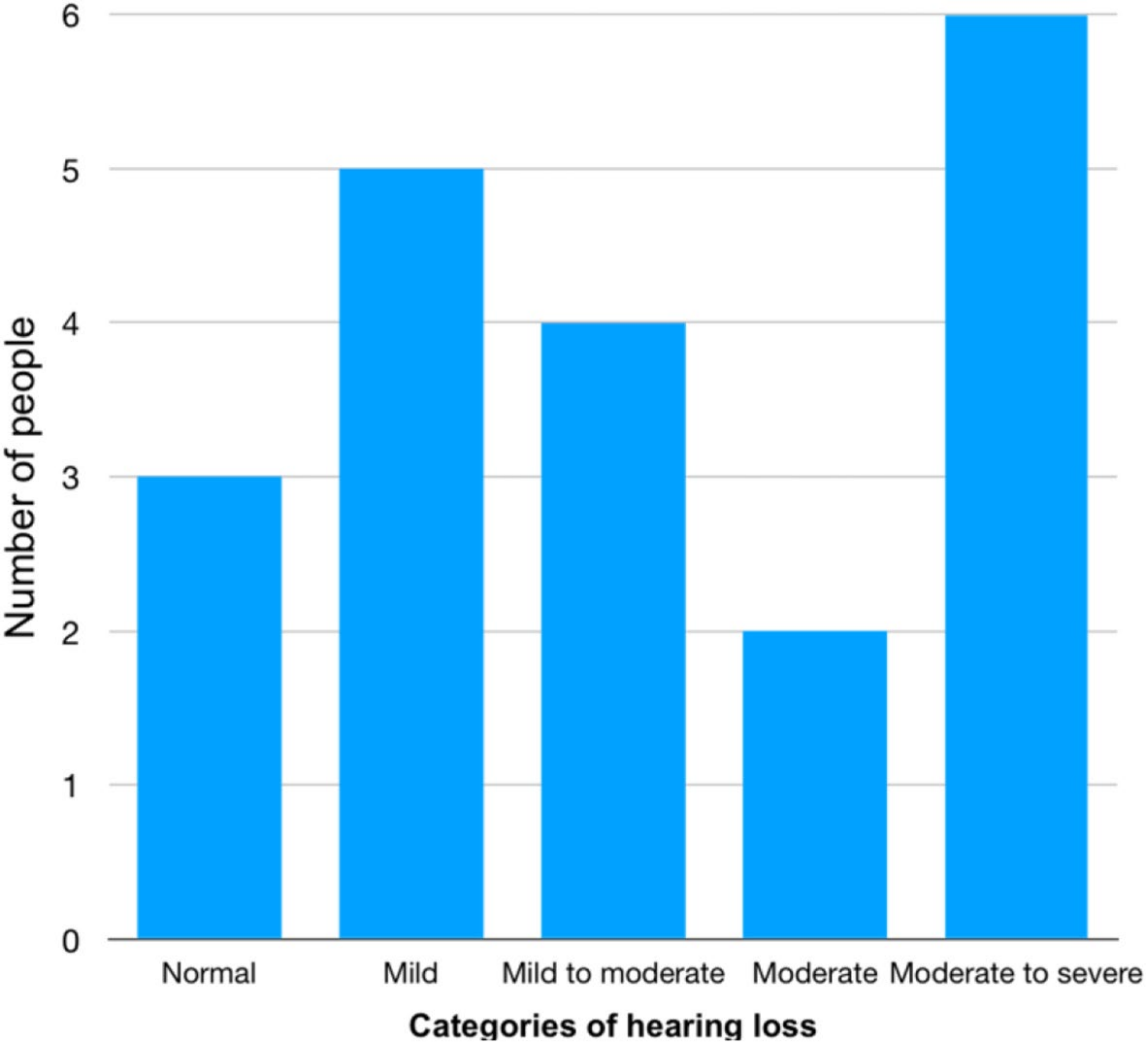
Categories of hearing loss for included patients

On audiology assessment, seventeen respondents (17/20, 85%) had at least a mild hearing loss detected. Categories of hearing loss are summarized in Fig. 2, with the ranges classified according to the WHO cut-offs [22]. At higher frequencies of at least 1 Hz, all patients had some degree of hearing loss detected. Nine respondents (9/20, 45%) had a significant amount of wax. Eight (8/20, 40%) reported subjective hearing loss at the time of audiology assessment. There was no significant correlation between self-reported hearing loss and objective hearing loss in this study, X^2^ (1, *N* = 20) = 2.81, *p* = 0.094. Just six patients (6/20, 30%) met the speech discrimination score of 100% in at least one ear, a marker of functional hearing ability. The mean MMSE for patients with moderate to severe hearing loss was 25.8, versus 27 for those with normal or mild to moderate hearing loss (p = 0.12), showing a tendency towards better results on cognitive assessment for the group with better hearing.

The first part of the patient survey looked at self-reported hearing issues, and assessments and interventions for hearing prior to their memory clinic assessment (Table 2). Within this survey, twelve patients (12/20, 60%) reported noticing hearing loss prior to their audiology assessment, a higher number than those who had reported it at the time of the memory clinic and audiology assessment. The majority of patients (15/20, 75%) felt a hearing test was an important part of their memory assessment, but just seven (7/20, 35%) identified a connection between hearing loss and memory problems.

**Table 2:** Patient survey questions and responses

n = 20	Yes	No	Unsure
Q1. Prior to attending the memory clinic, did you think that you were suffering from hearing impairment?	8 (40%)	12 (60%)	0
Q2. Prior to attending the memory clinic, had you noticed difficulties following conversations when there was background noise? e.g. other people talking, music, TV	12 (60%)	8 (40%)	0
Q3. Had you ever had a hearing test prior to your first attendance at the memory clinic?	7 (35%)	13 (65%)	0
Q4. Did you routinely wear a hearing aid prior to your first attendance at the memory clinic?	0	20 (100%)	0
Q5. Was it explained to you why a hearing test formed part of your assessment at the memory clinic?	6 (30%)	8 (40%)	6 (30%)
Q7. Do you think that there is an association between hearing problems and memory loss?	7 (35%)	9 (45%)	4 (20%)
Q8. Following your hearing test, was it recommended that you wear a hearing aid?	8 (40%)	12 (60%)	0 (0%)

Eleven patients (11/20, 55%) had hearing deficits sufficient to necessitate use of a hearing aid following their audiology assessment, with eight (8/20, 40%) referred for a hearing aid, and a further three patients who were offered referrals, but declined due to financial constraints.

Only five patients of those recommended for a hearing aid (5/11, 45%), had one available for use at the time of the survey, and of those, one never wore it, citing discomfort. An average of 11.2 (range 2–17) months had passed between their audiology assessment and the survey being carried out. Only one patient wore their hearing aid regularly, one occasionally and two in particular situations, specifically when watching TV and when leaving their home. One patient reported a subjective positive impact on their memory from wearing a hearing aid, with two reporting a positive impact on their participation in hobbies and past-times and one reporting a positive impact on their social life and relationships. Of note, none of those who wore their hearing aids reported any negative impact as a result of doing so. When patients were asked about their overall experience, the majority of responses were positive. The main themes identified related to the amount of information or results received, and to the process itself. With regard to the information or results received, ten patients (10/20, 50%) made positive comments about the amount of information, results and recommendations—“I got plenty of information” and “Things were explained well” were some of the positive comments in this area. However, not recalling whether information was given or whether it was adequate, was another recurring theme. This is illustrated by comments made by two patients; “I’m not sure if I got any information on the results, maybe I was told on the day” and “I think I got enough information, that I can remember”. A further two felt that the amount of information given was inadequate; “I don’t remember getting the results” and “I got very little information, they could have given me more”.

Twelve patients made comments on the process itself, all of which were positive. One theme in this area was the sentiment that it was an important part of their overall care or one that they felt fortunate to have had the opportunity to engage with, illustrated by comments such as “I’m happy to have had the hearing test” and “I’m lucky to be looked after”. Other comments in this area were on the logistics and domains assessed during the audiology assessment, with comments such as: “It was a very extensive evaluation” and “more thorough than I expected”.

Of the thirty patients with MCI contacted at the beginning of the study, nine (30%) had forgotten having had an audiological evaluation at all, a relevant group in the context of examining the patient experience. The comparison between this group and those who could recall their audiology assessment is summarised in Table 3. They tended to be older than the group who could recall their audiology assessment and tended to have a lower MMSE score. Two patients (2/9, 22%) were referred for hearing aids, compared with eight (8/20, 40%) in the group that could recall having had an audiology assessment, which may account somewhat for the recall difference between the groups. A longer period had also elapsed since their audiology assessment took place, at a mean of 13 months, compared with 12 months (p = 0.19) for those who recalled their audiology assessment. Although not statistically significant, they may give an indication of some of the factors that should be considered when assessing which patients with MCI are less likely to retain important information regarding their diagnosis and risk factor modification.

**Table 3:** Differences between patient who could and could not recall their audiology assessments

n = 20	**Patients who recalled audiology assessment**	**Patients who had forgotten audiology assessment**	**P values**
**Mean age (years)**	73	76	p = 0.19
**Mean MMSE**	26.35	25.57	p = 0.29
**Percentage referred for hearing aid**	40%	22%	p = 0.35
**Mean time elapsed between audiology assessment and survey (months)**	12	13	p = 0.19

## Discussion

Patients with MCI attending the memory clinic in this tertiary referral centre who agreed to audiology assessment were satisfied with their experience of having an audiological evaluation, and rated it as an important part of their memory assessment. With the evolution of the link between hearing loss and memory disorders, establishing that this pathway is acceptable to patients is important, and may lead to development of a framework for integration of audiology assessment into the practices of memory clinics. Further studies may focus on those who declined audiology assessment and their perception of this pathway.

Audiology assessment and intervention for hearing loss are clearly important in populations with cognitive disorders. Hearing loss has been established as a major mid-life risk factor for dementia [2] and is associated with a more rapid decline in cognition [23]. Hearing aids can potentially have a positive impact on cognition [13, 17, 18], as well as quality of life and behavior [10] in people with cognitive impairment. In addition, this study demonstrated a high prevalence of otological problems including wax and hearing loss, which would otherwise have gone undetected. The numbers who are non-compliant with hearing aid recommendations in this study is significant, in keeping with previous literature [15]. The existing pathway to diagnose and quantify hearing loss is futile in the absence of access to the recommended intervention for all patients. The small number of patients surveyed who regularly wear their hearing aids endorse their positive impact, with all reporting a positive impact on at least one domain of their lives.

Patient recall of the information provided around audiology assessments was poor, with 30% of patients contacted unable to recall having had an audiology assessment. Significant numbers also stated that the rationale behind having a hearing test as part of their memory clinic assessment was not explained to them. Furthermore, the minority of patients surveyed identified a link between hearing loss and memory disorders. There was no significant correlation between self-reported hearing loss and objective hearing deficits, in keeping with previous studies [7] and underlining the importance of audiology screening based on cognitive diagnosis rather than self-report in individuals with cognitive impairment.

The group of patients included in this study are a somewhat vulnerable cohort, with high levels of medical co-morbidity and polypharmacy. The majority of patients had more than one significant cardiovascular comorbidity, reflecting the high population prevalence of these conditions [24]. Risk factors for dementia cannot be addressed in isolation; a wide-reaching approach and tailored services are needed to optimise brain-health and quality of life. Communication and integration of different services providing assessment, risk factor modification and care of patients with cognitive impairment is vital.

### Limitations

This was a small-scale study in a single centre. Although all patients with MCI who agreed to audiology referral were included, this potentially omits patients who declined assessment as hearing was not a significant issue for them. Recall bias is evident, with a higher number of patients acknowledging subjective hearing loss at the time of the survey than did at the time of audiology assessment. For different patients, different time periods had elapsed between when the survey was administered and when their audiology assessment had been carried out, potentially excluding patients who may have been able to recall having had an audiology assessment at an earlier date. The range of time elapsed prior to the survey may also have impacted on the accuracy of answers to certain questions and opinions expressed. Data on educational attainment, as well as basic and instrumental activities of daily living was not available for all participants.

## Conclusions

Patients who participate in routine audiological evaluation as part of a memory clinic assessment are satisfied with this pathway. The amount of hearing loss detected highlights the importance of incorporating some kind of audiology screening into memory clinic pathways, especially given the impact that hearing loss can have on a patient’s risk of future cognitive decline, functional status and quality of life. Without such a pathway, hearing loss can frequently be overlooked and the opportunity to modify a patient’s dementia risk factor profile can be lost as a result. This needs be done in tandem with excellent communication and education about the links between hearing impairment and memory disorders, as well as a pathway whereby patients can easily access interventions.

## Supplementary Information

Additional file 1: (DOCX 17 kb)

## Data Availability

The data sets used and analysed during the current study are available from the corresponding author on reasonable request.

## Abbreviations

MCI: Mild Cognitive Impairment
WHO: World Health Organization
ENT: Ear Nose and Throat
MMSE: Mini Mental State Examination
