# Supplementary material for: Embedding audiological screening within memory clinic care pathway for individuals at risk of cognitive decline—patient perspectives
Source: BMC Geriatr. 2021 Dec 14;21:691. doi: 10.1186/s12877-021-02701-0 (PMC8672578; doi:10.1186/s12877-021-02701-0)
Supplement: Supplementary file 1 — Additional file 1: (DOCX 17 kb) [file 12877_2021_2701_MOESM1_ESM.docx]

**Appendix 1**

Hearing test survey

1. Prior to attending the memory clinic, did you think that you were suffering from hearing impairment?

- - Yes
  - No
  - Unsure

2. Prior to attending the memory clinic, had you noticed difficulties following conversations when there was background noise? e.g. other people talking, music, TV

- - Yes
  - No
  - Unsure

3. Had you ever had a hearing test prior to your first attendance at the memory clinic?

- - Yes
  - No
  - Unsure

4. Did you routinely wear a hearing aid prior to your first attendance at the memory clinic?

- - Yes
  - No
  - Unsure

5. Was it explained to you why a hearing test formed part of your assessment at the memory clinic?

- - Yes
  - No
  - Unsure

6. How important do you think having a hearing test was as part of your overall assessment at the memory clinic?

- - Important
  - Neutral
  - Low importance

7. Do you think that there is a association between hearing problems and memory loss?

- - Yes, there is an association
  - No association
  - Unsure

8. Following your hearing test, was it recommended that you wear a hearing aid?

- - Yes
  - No
  - Unsure

9. If it was recommended that you wear a hearing aid, do you wear it on a regular basis?

- - Yes, all of the time
  - Yes, regularly
  - Yes, occasionally
  - Yes, in particular situations
  - Never

If not, why not?

10. What impact, if any, has it made on your memory?

- - Positive impact
  - No obvious impact
  - Negative impact

On your social life and relationships?

- - Positive impact
  - No obvious impact
  - Negative impact

On your participation in hobbies and pastimes?

- - Positive impact
  - No obvious impact
  - Negative impact

On your mood?

- - Positive impact
  - No obvious impact
  - Negative impact

On your overall health?

- - Positive impact
  - No obvious impact
  - Negative impact

11. Do you have any other comments about your experience of having a hearing test as part of your memory clinic assessment?

Is there anything we could improve on? Do you feel you received adequate information about the test and the results?
